# "It's almost expected": rural Australian Aboriginal women's reflections on smoking initiation and maintenance: a qualitative study

**DOI:** 10.1186/1472-6874-11-55

**Published:** 2011-12-09

**Authors:** Megan E Passey, Jennifer T Gale, Robert W Sanson-Fisher

**Affiliations:** 1 University Centre for Rural Health - North Coast, School of Public Health, University of Sydney, Lismore, NSW, Australia; 2 School of Medicine and Public Health, University of Newcastle, Newcastle, NSW, Australia

## Abstract

**Background:**

Despite declining smoking rates among the general Australian population, rates among Indigenous Australians remain high, with 47% of the Indigenous population reporting daily smoking - twice that of other Australians. Among women, smoking rates are highest in younger age groups, with more than half of Aboriginal women smoking during pregnancy. A lack of research focused on understanding the social context of smoking by Aboriginal women in rural Australia limits our ability to reduce these rates. This study aimed to explore the factors contributing to smoking initiation among rural Aboriginal women and girls and the social context within which smoking behaviour occurs.

**Methods:**

We conducted three focus groups with 14 Aboriginal women and service providers and 22 individual interviews with Aboriginal women from four rural communities to explore their perceptions of the factors contributing to smoking initiation among Aboriginal girls.

**Results:**

Four inter-related factors were considered important to understanding the social context in which girls start smoking: colonisation and the introduction of tobacco; normalization of smoking within separate Aboriginal social networks; disadvantage and stressful lives; and the importance of maintaining relationships within extended family and community networks. Within this context, young girls use smoking to attain status and as a way of asserting Aboriginal identity and group membership, a way of belonging, not of rebelling. Family and social structures were seen as providing strong support, but limited the capacity of parents to influence children not to smoke. Marginalization was perceived to contribute to limited aspirations and opportunities, leading to pleasure-seeking in the present rather than having goals for the future.

**Conclusions:**

The results support the importance of addressing contextual factors in any strategies aimed at preventing smoking initiation or supporting cessation among Aboriginal girls and women. It is critical to acknowledge Aboriginal identity and culture as a source of empowerment; and to recognise the role of persistent marginalization in contributing to the high prevalence and initiation of smoking.

## Background

Addressing tobacco smoking among Australia's Aboriginal and Torres Strait Islander population is critically important to reducing the excessive burden of disease borne by this group [1]. Tobacco smoking causes or exacerbates lung cancer, chronic obstructive pulmonary disease, asthma, other cancers, cardiovascular disease, pregnancy complications and low birth weight as well as numerous other conditions [2]. It is the largest preventable cause of morbidity and mortality for Aboriginal and Torres Strait Islander people [1]. Despite declining smoking rates among the general Australian population, rates among Indigenous Australians remain high, with 47% of the Indigenous population reporting daily smoking compared to 20.9% of the non-Indigenous population [3]. Smoking rates are similarly elevated among the Indigenous peoples of New Zealand, Canada and the United States [4-6].

Socio-economic disadvantage and associated stressors are recognised as drivers of smoking internationally [7-9]. While it is acknowledged that the socio-economic disadvantage suffered by many Indigenous Australians contributes to the high prevalence, the rates of smoking are higher for Indigenous than non-Indigenous Australians within socio-demographic groupings. For instance stratifying by employment status, education or income, Indigenous Australians are almost twice as likely to smoke as their non-Indigenous counterparts [6]. Similarly in New Zealand, within all age, gender and socio-economic groupings, the prevalence of smoking among Maori is higher than among non-Indigenous New Zealanders [10] indicating that factors beyond socio-economic differentials are contributing to differences in smoking behaviour. However, there is still limited empirical evidence to increase our understanding of the processes driving this differential at the local level or the best approach to reducing it.

Among Australian Aboriginal peoples, the experience of colonisation has contributed to the use of tobacco through both the social consequences of colonisation and the introduction of tobacco [11-13]. Tobacco was used traditionally in northern Australia, through trade with Macassan fisherman, but its availability was seasonal and its use regulated through social control mechanisms [11,12,14]. In south-eastern Australia, tobacco was not used traditionally [11]. Following European colonisation tobacco became more widely available throughout Australia and was included in rations on missions and cattle stations [11]. Thus, although the traditional patterns of use varied, the coming of Europeans greatly increased supply, and created widespread use of tobacco among Aboriginal peoples. Social consequences of colonisation contributing to the high rates of smoking include the experience of land dispossession, loss of language, culture and social systems [15], and subsequently becoming a marginalised group with considerable socio-economic disadvantage and high levels of stress [11-14,16]. Normalisation of smoking in Aboriginal communities and the cultural value placed on maintaining interpersonal relationships through reciprocity and sharing of resources are also considered to contribute to the high prevalence of tobacco smoking [11-14,16].

Smoking is the most important preventable cause of foetal and perinatal mortality in western countries [17]. Indigenous births in Australia are characterised by a higher proportion of premature births, small for gestational age babies and low birth weight babies relative to births to non-Indigenous women [18,19]. Smoking is an independent predictor of these outcomes among Indigenous women [2,19,20]. In 2007 the prevalence of smoking among pregnant Indigenous women in Australia was 51.8% compared to 14.8% in non-Indigenous women[18]. In the same year, 12.5% of Indigenous births were low birth weight, and 13.7% were premature, compared with 5.9% and 7.9% respectively for non-Indigenous births [18].

Addressing smoking during pregnancy requires an understanding of factors influencing being a smoker at the beginning of pregnancy, and those influencing cessation or continuation during the pregnancy. A large body of work has explored the factors influencing tobacco initiation and identified the role of peers [21,22], the social and physical environment [23-25], and parental behaviour [26-28] as influential in uptake of smoking. Qualitative research with adolescent girls has emphasised the role of smoking in self-definition, making social distinctions and acquiring status [29]. However, there has been surprisingly little work exploring smoking initiation among Aboriginal people in Australia.

In a nation-wide consultation process on Aboriginal smoking more broadly, Lindorff identified the importance of children wanting to belong and be accepted by their peer group as contributing to smoking initiation [12]. In remote Northern Territory communities, family influences and intergenerational transmission were critical in shaping youth smoking behaviour [14]. A recent Western Australian study with both Indigenous and non-Indigenous adolescents found that smoking status had little relevance to friendship selection but that lower socio-economic and Aboriginal adolescents reported more peer pressure to try smoking [30]. Smoking rates are also elevated among Native American [31,32] and First Nations Canadian [33-35] youth, relative to non-Indigenous youth in North America. Correlates of smoking among Indigenous North American adolescents include death or loss of a friend or family member and other stressful life events [36], and maternal smoking during and after pregnancy [37]. Greater academic orientation has been identified as having a protective effect [36]. Indigenous youth have also been found to have greater exposure to tobacco in the home environment [33], to have higher access to cigarettes and greater exposure to smoking peers than other groups [32].

While there is a growing body of research on Indigenous Australian smoking, particularly in remote communities, we are unaware of any work specifically examining the social context of smoking or its initiation by Aboriginal women in rural Australia, where 43% of the Indigenous population live [38]. Given the importance of contextual factors in shaping smoking behaviour, an understanding of the experiences of rural Aboriginal women is critical to developing effective approaches to reduce their tobacco use. In rural Australian towns, the Aboriginal population is generally a minority group living among a dominant non-Indigenous population, grouped together on housing estates and in Aboriginal communities. Due to earlier government policies including forced relocation, Aboriginal people from many parts of Australia live alongside the traditional owners. This contrasts with remote Australian communities, where Aboriginal people are in the majority and are more likely to live on traditional lands, maintaining strong kinship ties and experiencing less cultural disruption. In this study we explore the factors contributing to smoking initiation among rural Aboriginal women and girls and the social context within which smoking behaviour occurs.

## Methods

This exploratory qualitative study involved rural Australian Aboriginal women and service providers in focus groups and semi-structured interviews, to explore their perceptions of social and environmental factors contributing to the high prevalence of smoking; and the role of individual, family and community influences on smoking initiation. The approach was informed by a belief that the social reality for our participants could only be understood through their interpretations of their experiences[39]. The approach was also influenced by Indigenist methodology, which emphasises the importance of relationality, reciprocity and respect[40,41]. This was manifest through a collaborative approach with the local Aboriginal community from the inception of the study to its completion. The findings presented here are part of a larger project addressing smoking and smoking cessation during pregnancy. The study commenced in June 2007, with data collection between September 2007 and February 2008. Ethical approval for this study was obtained from the North Coast Area Health Service Human Research Ethics Committee and the Human Research Ethics Committee of the University of Newcastle.

Initial consultations were held with the local Elders Council. A community reference group (CRG) was formed on their advice, to guide the study and ensure it was conducted in a culturally secure manner [42]. The CRG was composed of five Aboriginal women from the community and five female Aboriginal Health Workers working in maternal and child health. Aboriginal Health Workers are a specific category of Australian health professional. They work in a variety of roles and settings to help bridge the cultural gap between Aboriginal people and the western medical system. The CRG provided advice on the research questions, study design, participant recruitment, interpretation and reporting of results, and met six times during the course of the study. The research team was comprised of non-Indigenous academics and a female Aboriginal community researcher. For this project we collaborated with the community Midwife and Aboriginal Health Worker from the local Aboriginal Maternal and Infant Health Strategy (AMIHS) antenatal team.

### Study site

The research was undertaken in a coastal, river region of NSW. It is a rural area, with numerous small communities scattered among towns and regional centres, similar to other coastal areas of Australia. The area experiences considerable socioeconomic disadvantage, with the highest rates of people receiving unemployment benefits (7.7%) and disability or sickness benefits (13.2%) of any Health Area in NSW [43]. Within this area, the AMIHS team provides antenatal care in several communities, including a regional centre of 45,000 people, in which the Aboriginal population is clustered within two suburbs, a town of 17,000 people, a village of approximately 500 people and several Aboriginal communities located on community owned land. Aboriginal people, who make up approximately 3.7% of the population in the area [44], come from across Australia and are not necessarily related to each other.

### Data collection

#### Focus groups

Three focus groups were held prior to the interviews. Focus groups were used initially to explore the range of issues and perspectives that could be elicited through this interactive approach to data collection[45]. They were also useful in understanding the most appropriate language and colloquial expressions to use, and to assess the acceptability of discussing potentially sensitive issues prior to the individual interviews. The first focus group was held with six Aboriginal Health Workers and one non-Indigenous midwife (the "clinician's group"). Most of the participants were members of the local Aboriginal community and all provided services within the community and had extensive knowledge of both health behaviours and factors influencing behaviour locally. It was felt that their experiences in providing health care might provide additional valuable insights into smoking behaviour not identified through discussions with other participants. The second focus group was with older community women (over 25 years) and the third with younger women (less than 25 years). These two groups were held separately as their experiences and perspectives were likely to differ, and each may feel less constrained in separate forums. The participants were identified by members of the CRG as being articulate and knowledgeable members of the community. They included smokers and non-smokers, and women who were mothers and grandmothers and girls who had not had children. The focus groups, lasting approximately two hours, were conducted by two female researchers - one Aboriginal (JG) and one non-Indigenous (MP).

#### Semi-structured interviews

Following the focus groups, individual semi-structured interviews were held with 22 other local women who were either currently pregnant or had given birth within 12 months. Individual interviews were important for understanding individual views and experiences and for capturing women's personal stories[46]. As smoking during pregnancy was the focus of the larger study, it was important to recruit women with recent experiences of pregnancy. Interviews were conducted by the Aboriginal researcher at sites selected by the women to maximise their comfort with the interview. These included parks, riverbanks, a skatepark and private homes.

#### Recruitment

Women were recruited by the antenatal team or members of the CRG, who explained the study then provided contact details of interested women to the Aboriginal researcher. She contacted the women, provided written information and arranged the woman's attendance at either a focus group or an individual interview as appropriate. Purposive sampling to maximise variation[47] ensured inclusion of smokers and non-smokers; women from a variety of locations; and with a range of ages and parity. Thematic saturation was achieved in relation to responses from women who smoked and from those who had quit during pregnancy. We were unable to recruit sufficient non-smokers to achieve saturation regarding never smoking or successful permanent quitting. All non-smoking clients of the antenatal service at the time were interviewed. All participants provided written consent.

#### Topic areas

Topics explored in both the focus groups and individual interviews were identified from a review of the literature on smoking initiation and Indigenous smoking; and through team and CRG discussions. A topic list guided the process, but respondents were encouraged to talk freely, telling their stories in their own way. The flexible interview guide, allowed exploration of additional issues raised by women within the interview and in subsequent interviews. Women were encouraged to discuss both their own experiences and their observations and perceptions of the behaviour of others. Topics included:

• Social and environmental factors: the social, cultural and physical environment, and people's perceptions of how this influenced smoking behaviour; social norms and expectations related to smoking; general perceptions of smoking; prevalence of smoking; acceptability of smoking generally and for different groups or circumstances;

• Smoking initiation: experiences with starting smoking including the circumstances of starting; factors influencing decisions regarding smoking; role of others in starting smoking; views on why girls start smoking; access to and sources of cigarettes.

### Data management and analysis

Focus groups and interviews were digitally recorded and transcribed verbatim. Each transcript was reviewed for accuracy then offered to participants for review and comment. A content analysis process was used to reveal codes relevant to themes related to the study topic and to identify additional themes[48]. Transcripts were read repeatedly by the first author to familiarise herself with the data. Initial codes were identified and the findings related to these codes summarised and presented to the CRG for discussion and further refinement. Initial codes either related specifically to the interview topics or were derived from the data. Transcripts were then line-by-line coded, with the codes revised from the CRG meeting, using N-Vivo 7. Further codes were developed as additional concepts were identified. The data were organised into themes based on patterns identified, with further review of themes and their relationships to each other and to various stages of smoking behaviour. Comparisons were made between responses from the focus groups and the individual interviews and between women based on the sampling approach (age, parity and location). The results were presented to the CRG at intervals throughout the analysis, to confirm interpretation and further elaborate issues.

## Results

Fourteen women participated in the focus groups: seven in the clinician's group, three in the older women's group and four in the young women's group. The focus group participants included smokers, ex-smokers and women who had never smoked. Some were mothers of young children or of teenagers; others were grandmothers or girls without children. The Aboriginal Health Workers came from both government and non-government services, and had all been working in the field in excess of 10 years. The characteristics of the 22 women interviewed individually are shown in Table 1.

**Table 1:** Characteristics of women interviewed (n = 22)

Characteristic	n	Mean (range)
Age (years)		24.9 (17-41)

Educational status		
Year 10 or less	15	
Year 11	3	
Certificate/diploma	3	

Income source		
Employment	2	
Welfare	17	
Other	3	

Pregnancy Status		
Pregnant now	13	
Recently pregnant	9	

Parity		2.1 (0-7)

Tobacco smoking		
Never smoked	2	
Ex-smoker	1	
Quit during pregnancy	8	
Cut down during pregnancy	6	
No change	3	
Increased	2	

Data from the focus groups and individual interviews were similar in providing individual, personal experiences, with the exception that the clinician's focus group also discussed issues from the perspective of people providing community services. While there was some variation in responses between older women and younger women, as reported here, there was no clear pattern by parity or location. Women who had never smoked reported different personal experiences to those who smoked, but their views of the factors influencing smoking behaviour were similar to those who smoked.

Results on environmental factors influencing smoking are presented first, to provide the context for smoking behaviour, followed by the results specific to smoking initiation. In the following sections we elaborate on the four themes which provide insights into the social context in which Aboriginal girls in these communities start smoking: colonisation and the introduction of tobacco; separate Aboriginal social networks and normalization of smoking within these networks; disadvantage and stressful lives; and the importance of maintaining relationships.

### Contextual factors contributing to the high prevalence of smoking

#### Colonisation and introduction of tobacco

European colonisation created extensive disruption to Aboriginal society including dispossession of traditional lands, movement of people, removal of children, loss of traditional lifestyle and introduction of tobacco and alcohol[49]. The introduction of tobacco by Europeans together with other lifestyle changes, were raised by participants in each of the focus groups and by some older women in interviews. People no longer live traditionally and with the lifestyle changes, women reported less respect for elders and the loss of other traditional behavioural constraints. It was recognised that traditional smoking had occurred in other parts of Australia, but in the context of ritual and ceremony, with limits around smoking which no longer apply. Tobacco is now readily available with few social proscriptions on its use.

*So there was things that were in place, there were controls put in place, whereas now we have no controls. Stuff comes in, anybody can smoke anything,... there's no rituals....*.

Clinician's focus group

*We never had smoking from our previous ancestors, we never had smoking. It has a lot to do with Western society bringing it,...... If it was never introduced, same as alcohol, then maybe we probably wouldn't have people smoking, yeah........ It's changed our whole way*.

41 year old ex-smoker, mother of 6

#### Social networks and community norms

Despite mostly living in towns with large populations of non-Indigenous people, the Aboriginal population tends to remain relatively socially separated from the non-Indigenous population. This, combined with different social norms regarding smoking creates a situation where many people have limited interaction with non-smokers. The participants described very separate Aboriginal social networks embedded within the broader community. The CRG discussed this issue extensively and confirmed that local Aboriginal people mainly interact with other Aboriginal people. They indicated that this is partly due to a preference for socialising with relatives and others who share similar culture, values and history, and the strong social support gained through these networks. However, it was also thought to be due to experiences of exclusion or marginalisation from mainstream society in regard to participation in work, school and other civic activities. While some Aboriginal people in the community have jobs, many do not. For these people there are limited opportunities for interaction with the broader community's social institutions and people, and the extent of social separation is greater.

The participants reported a high prevalence of smoking within the Aboriginal networks which then leads to a perception that smoking is normal and part of Aboriginal identity. The limited interaction with the broader community also limits exposure to non-smokers and to changing attitudes to smoking. Women reported that smoking was generally acceptable provided household rules around smoking outside or away from children or sick people were respected.

*When you don't smoke you stand out a bit, you feel a bit odd.... Cause everyone around you smokes. Everyone that I know smokes. There is not one person that I know in [town] who doesn't smoke. So like, it is a bit hard [not to smoke]*.

24 year old smoker

*I reckon about 95 per cent of Koori people smoke........Every household just about like I say, 95 percent have got people smoking*.

33 year old smoker

#### Disadvantage and stressful lives

Women in these communities suffer from excessive levels of daily stress as a result of numerous social, emotional and financial pressures. High unemployment and associated poverty lead to difficulties finding affordable housing. Overcrowding is common as extended family and friends frequently stay for lengthy periods, exacerbating financial and social pressures. Relationship difficulties and loss of family members through death or removal of children further contribute to high stress levels. Participants considered that these frequent daily stressors contributed to the high prevalence of smoking and other substance use, as these were used to provide temporary relief.

Many participants spoke about grief and loss in their everyday lives. During the period of data collection there were many deaths in the Aboriginal community, including two of the participants. The frequent and often premature deaths mean that funerals become an everyday event, which both bind the community together and cause considerable grief. Several of the women interviewed had lost one or more of their parents as children, either through death or being removed from parental care. Others had experienced removal of their own children, and there were many stories of physical and sexual abuse of children. Many women had also experienced violence and/or sexual abuse as adults.

*I was only with this bloke for 2 months - I got kicked out of my place, he had holes in my walls. I just left everything all my possessions,... and went to where I could get support from family..... Now back on my feet now all I need to do is get a place*.

and

*I was molested by the same people that looked after me, my mum's sister's husband. My mum died when I was like 8 and we didn't pretty much know dad. He died.... I nearly tried to kill myself*.

Interviewer: *And what did your aunty do...?*

*She didn't know nothing. I didn't tell her because she was dying of cancer...... I kept strong for her, held it in you know*.

28 year old smoker

They also described experiences of perceived racism including difficulties finding housing and jobs, police harassment and being confronted with negative stereotypes. They felt this reflected the lack of interaction between Aboriginal and non-Indigenous people. The experience of racism, combined with grief and loss, ongoing violence, high unemployment and lack of housing, created high levels of stress in the women's lives on an ongoing basis.

#### Maintaining relationships and sharing

Relationships with other members of the extended family and community are highly valued in Aboriginal culture and may be given priority over individual needs. Maintenance of these relationships involves considerable social interaction and reciprocity with obligations to share time and resources, including food and accommodation. Participants described extensive and complex relationships, both within the extended family and the larger Aboriginal community, with family and friendship networks extending over large geographic areas. Obligations to give cigarettes to others were described by nearly all participants and many also described an obligation to accept offered cigarettes. Sharing a cigarette and having a yarn, was an important social activity, contributing to a sense of belonging to the Aboriginal community.

While providing cigarettes to others was sometimes resented due to their high cost, they were seldom refused. Older women mentioned family arguments resulting from the obligation to share cigarettes, and themselves resorting to hiding part of a packet in order to reduce this obligation. The willingness to share appeared more common among younger women who possibly had fewer financial responsibilities or less established nicotine dependency.

*Don't believe in arguing over smokes, you got it, you give it, you don't, you can't*.

17 year old smoker

*They'll ask (partner) and they'll get him on his own when I'm not around.... When he gives out smokes that's when I get shitty. Don't give them smokes, come back here and gives us smokes first and then we'll give you a smoke, 'cause we ain't rich.....*.

28 year old smoker

### Initiating smoking

The 20 ever-smokers among the women interviewed individually reported starting at a mean age of 15 years (range 12-21) and becoming regular smokers at a mean age of 16 years (range 12-21). Every participant expressed the view that children were starting smoking at a younger age now than previously, with many children starting at age 12 or 13, and some starting younger.

Within the context of separate Aboriginal social networks, considerable disadvantage, and a high prevalence of smoking, young girls use smoking to attain status and assert their group membership and Aboriginal identity. Family and social structures, while providing strong supports, limit the capacity of individual parents to influence their children not to smoke, and provide children with ready access to cigarettes. The experience of marginalization is considered to contribute to limited opportunities and aspirations for young people, leading them to seek pleasure in the present rather than having goals for the future. We elaborate on these themes below.

#### Peer influences and needing to belong, smoking as status

Peer influences were manifested through girls wanting to be like others, socialise with them and belong to their group rather than through overt peer pressure. Transition from primary school to high school was a particularly vulnerable point where girls negotiated new relationships and used smoking as symbolic 'adult' behaviour to gain status and be accepted. In describing their own experiences, women emphasised the role of Aboriginal peers, particularly cousins and friends, in influencing them starting smoking. Most reported having their first cigarette because of the social aspect of smoking, wanting to fit in with their friends and belong to the group. Some women reported having their first cigarette while drinking with friends or family and that smoking was just part of the whole social process. While most women did not feel pressured to start smoking, a few did describe feeling pressured, and despite not wanting to, smoking in order to belong. However, the emphasis expressed by the vast majority related to the desirability of belonging and participating in the social activities of the group, more than a pressure to smoke.

*Friends, friends. Because when I first started smoking my influence was my friends were smoking and I thought well they're smoking so I've got to start smoking. Yeah, so it's more a friends thing, and sometimes it can be you see your parents do it as well*.

24 year old, started age 15

Although they described some mimicry of smoking and experimentation with cigarettes among very young children, regular smoking among primary school children was uncommon and children would often tell adults they shouldn't smoke. However, once children went to high school they rapidly took up the habit. Many participants felt that the main motivator among young high school students was to appear older, 'cooler' and to be accepted by older adolescents in the school. The transition to high school was difficult, particularly for children who didn't engage well with the school system, and smoking was thought to be used by children to attain higher or 'adult' status.

Participant 1 *As soon as they hit high school, that's when they're into it straight away*.

Participant 2 *Some of the young ones, like some young ones at home, that hang around with high school ones... and they think they're real deadly and they'll give them a couple of draws but they might not full on start, but once you get to high school - its gone! *

Young women's focus group

*When I was a young girl, I was in this group and most were older than me and it was like a cool thing, all your friends are doing it. I felt left out so I just started asking. I thought I was a big woman [laugh]. I was only 13. I didn't like it though *

33 year old smoker

#### Development of identity

Growing up in a marginalised social group where smoking is a normalised and common behaviour, children are socialised to smoking from an early age and may incorporate it into their sense of identity. Internalisation of a smoking identity as part of their overall identity was described by many women and discussed by the CRG. For children growing up in Aboriginal families where smoking is common, smoking becomes incorporated into their identity and becomes blurred with their Aboriginal identity. The women felt that there was an unspoken expectation that children would become smokers.

*Your mob smokes, so it's in your blood to do it*.

19 year old smoker

*I dunno it's almost expected isn't it like?... I dunno, I never thought about that, but yeah. Probably it's almost expected*.

33 year old smoker

#### Social structure and parenting

The extended family structure and communal network among Aboriginal people are strong in both the geographically isolated communities and within the larger towns. Many households are large and fluid, with people coming and going, and extended family members of multiple generations living together for lengthy periods. Raising children is often communal, with extended family members or friends frequently caring for children. The social structure of Aboriginal communities and shared child-raising, when combined with the high smoking prevalence, was identified as contributing to children initiating smoking. Individual parents have less control over their own children's behaviour, and there are fewer clear behavioural limits with children frequently exposed to many people smoking.

*[People start smoking] younger in the Aboriginal community, because they're all around each other.... all their family is one big family.... all together.... Most of the parents out there don't teach their kids or it's other people doing it for them. It's other people because mainly someone's always watching them*.

23 year old smoker, mother of 2

Women expressed paradoxical views regarding parental ability to influence their children's smoking behaviour. Although most women felt that the high prevalence of smoking among their parents and extended family had been influential in normalising smoking and making it more acceptable for them to start, they did not think that parents could influence children not to start smoking. Only one woman thought she would be able to influence her children not to smoke because neither she nor her partner smoked. Most women who were parents were resigned to the idea that their children would smoke.

*Yeah I always think to myself I don't want my boys to smoke cigarettes... but like if they're going to do it then they're going to do it. I can't stop them from doing it. I'll try, but I know I'm not going to be able to*.

24 year old smoker, mother of 4

When asked where children obtained cigarettes the smokers reported that they had obtained cigarettes from friends and cousins, or by taking them from adults' packets. The high prevalence of smoking, combined with the highly connected communal structure meant there were many possible sources of cigarettes. No one was aware of shops which sold cigarettes to minors, but some reported adults buying packets for children, or giving them single cigarettes, particularly if they were related to them and felt obliged to share their cigarettes. Others reported that adults were less likely to give children cigarettes now than in the past, due to increased awareness of the problems with smoking.

#### Lack of opportunity and present orientation

Limited opportunities for adolescents in rural towns and villages are exacerbated by marginalisation and felt more profoundly by disadvantaged groups with limited resources. Lack of opportunities may create boredom, limit aspirations and lead to a focus on immediate gratification. Participants believed that limited opportunities for Aboriginal children and adolescents in the region contributed to early initiation of smoking. In smaller communities, lack of local sports facilities combined with poor public transport, made it very difficult for children to engage in sports or other activities. The clinician's focus group and some older women also thought that lack of employment prospects resulting from limited social connections and racism played a role, as it inhibited the ambitions of high school children. These children then perceived little benefit from applying themselves in school and had few aspirations for their future. The clinicians reported difficulty motivating children and adolescents, as it was difficult to demonstrate success.

*How are we supposed to stop people from having anything like that when, you know, it's hard to even get them to keep going to school....... when they're closer to leaving school age, and they know there's nobody in their family or anybody has got a job..*.

Clinician's focus group

The boredom and lack of routine among unemployed adolescents who had left school also contributed, with some young people living hedonistic lifestyles with little concern for the future. The participants in the young women's focus group expressed an orientation toward pleasure rather than concern for their future health.

Participant1 *That's how you think - I'm going to die anyway, I might just do what I'm enjoying*.

Participant 2 *Like we gunna die of something*

Participant 3 *Yeah, instead of thinking 'Oh, I could give up and go and have a really good*, *happy, healthy life and live longer'....... Like you don't really think that way, you just think I enjoy this, I'm going to do it whatever*.

Young women's focus group

#### Protective influences

Anti-smoking parental advice and role modelling; and success in school, sports or other activities provided a protective effect from early smoking initiation for girls. Involvement in competitive sports gave girls an incentive to keep fit, friends who were non-smokers and disapproved of smoking, and alternative ways to gain acceptability and status. Several women reported only initiating smoking after discontinuing competitive sports, and consequently had started later than the other participants. The women who had continued their education to year 10 or beyond had also started smoking later than women who left school earlier.

*Sport tends to help them. If they're very into sport that usually will help them find another avenue, and also other non-smokers to go with. So it's not cool then. They might say they're bloody stupid or something*.

Clinician's focus group

*I started when I was 21.... I wish I was still fit, that I didn't start smoking....because I never was interested in smoking, like I wasn't even really into boys....I was worried about school and my sport*.

24 year old smoker

The two participants who had never smoked had been involved with sports, and one continued to play several sports. One also expressed concern with her health and emphasised the importance of her family influences - neither of her parents smoked and they had educated their children about the dangers of smoking, none of whom took up smoking. She described warning her own children about the dangers of smoking, and trying to ensure they weren't exposed to other people's smoke.

*I didn't like the stuff. And my parents made us aware of it growing up. They just, you know told us if you want to smoke you know it's bad for your body, bad for your lungs and it's better, best to be clean*.

25 year old, never smoked

## Discussion

We used qualitative methods to explore Aboriginal women's understandings of the social context of smoking and smoking initiation in rural communities in coastal NSW. The findings confirm the importance of the social and historical context in creating a pro-smoking environment with numerous external factors interacting with personal factors to drive the early initiation of smoking. Socio-economic disadvantage, housing shortages and high unemployment are associated with high levels of stress and contribute to the perpetuation of smoking. These findings support the importance of the social determinants of health in contributing to disparities in smoking rates. Further, they highlight the need to address these issues in strategies aimed at reducing Indigenous disadvantage [7,50,51].

The importance placed on maintaining relationships with the associated obligation to share resources was found to contribute to the high rates of smoking, consistent with earlier research [11-14,16]. Women derived clear social benefits from sharing cigarettes and a sense of belonging to the community through smoking. In their work in two remote Northern Territory communities Johnston & Thomas emphasised the value placed on reciprocity and sharing, and the role that cultural obligations play in smoking behaviour [14]. They argued that these cultural factors add another layer of complexity to social dynamics, resulting in qualitatively different drivers of smoking maintenance to those experienced by non-Indigenous disadvantaged groups with high smoking prevalence [14]. In our setting, Aboriginal people constitute a small minority of the population, and many come from other parts of Australia thus reducing kinship ties. However the emphasis placed on sharing and reciprocity by our participants and the sense of connectedness and belonging to the Aboriginal community remained strong. Relative to remote communities, where Aboriginal people are usually in the majority, the situation for Aboriginal people in rural and urban Australia differs. Here, the experience of marginalization and cultural disruption is likely to be more complex[52]. In this context, the importance of smoking in establishing and maintaining group membership and Aboriginal identity is different, and may include stronger assertion of group identity.

In our setting, the separation of Aboriginal and non-Indigenous social networks was perceived to be important in both initiation and maintenance of smoking. Analysis of data from the Framingham Study demonstrated that smoking behaviour spreads through social networks, with smokers becoming increasingly socially marginalised [53]. In Australia, the role that separate social networks play in smoking behaviour among Aboriginal Australians has received little attention. However, research on psycho-social constructs mediating health behaviours related to cardiovascular disease in rural Victoria is of interest[54]. The authors identified the relationships that Aboriginal people have with the broader society as an important issue. Marginalisation, restricted access to mainstream services and institutions and racial stereotypes helped create separate social networks and impacted on health behaviours [54]. An exploration of social capital and identity among Indigenous people living in metropolitan Brisbane found that respondents had strong bonding social capital through their connections with family and the broader Indigenous community[55]. However, "In the context of an oppressive history and experiences of ongoing racism and discrimination, a second world of bridging social capital remains elusive to many Indigenous Australians" [55]. These findings resonate with our own in articulating the importance of Aboriginal identity in social network formation, and the role social networks play in perpetuating smoking behaviour, including initiating smoking.

Our findings on the perceived influences on initiating smoking emphasise the role of Aboriginal social networks in determining peer groups and the importance of smoking in order to belong to a these social groups. Arnett (2007), in his critique of the concept of peer influence, has argued that a more appropriate concept is that of peer context, which takes account of friendship selection in determining peer influences [56]. In our setting, Aboriginal children are largely forming friendships with other Aboriginal children in a marginalized social network where smoking is normalized. The choice of smoking then reflects a desire to fit in and belong to this group, driven by "self-pressure" [57]. Johnston & Thomas emphasised the importance of intergenerational transmission of smoking behaviour in remote Northern Territory communities, with initiation almost universally influenced by family smoking practices, rather than peers [14]. Similarities with our own study include the strong environmental cues for smoking and ready access to cigarettes. Smoking as a means of asserting membership in the Aboriginal community was not identified as an important factor in the Australian remote setting. This may be because Aboriginal people form the majority in remote communities, and thus 'normalisation' of smoking works through a different mechanism. Shared care of children also limits the amount of control parents are able to exert over their own children's behaviour and their children's exposure to other people smoking. A similar problem has been described in Canadian First Nations reserve communities, where family obligations and crowded housing create difficulties for women in reducing children's smoke exposure [51].

In a review of the social context of smoking, Poland *et al *(2006) emphasised the importance of viewing smoking as a collective social practice, rather than just individual behaviour. They also highlighted the role of power relations in increasing social disparities in smoking behaviour; and the role of behaviour, including smoking, in constructing and maintaining a social identity [58]. In our study, lack of power and the experience of discrimination and marginalisation were perceived to work through several mechanisms to increase smoking initiation. These mechanisms included social separation from the broader society; a reduction in children's aspirations; and children feeling disconnected from their schools leading them to smoke to attain status. Smoking was also used to establish an identity and group membership. Dixon & Banwell (2009) recently reviewed the application of social theories to understanding the temporal spread of health risk behaviours, in particular the spread of smoking, among different social groups[59]. They drew on Bourdieusian concepts to explain the persistence of smoking among some disadvantaged groups and proposed a fifth phase to the smoking transition model proposed by Lopez *et al *[60]. In this fifth phase, successive cohorts of disadvantaged groups continue to adopt smoking despite widespread recognition of the harms. They suggest that intergenerational transmission of 'habitus', or way of life, combines with 'doubling' or copying others in the same social group, and the need for 'distinction' from other groups, to explain this continued initiation of harmful behaviours. Our own study provides some support for this, in that girls are thought to initiate smoking by adopting the behaviour of others within their social grouping, in order to assert their identity and membership of that group, and distinguish themselves from others.

### Limitations and strengths

This study is one of the first that has explored the social context of smoking and smoking initiation among Aboriginal people in rural Australia, shedding new light on this important issue. The study benefited from input from Aboriginal women from its inception, including having a respected Aboriginal woman conduct all interviews. Assistance and input from the CRG helped optimise cultural security and the exploration and interpretation of key concepts. Additionally, local community engagement was enhanced by providing verbal feedback and a brief written report which was widely circulated.

The findings from this study must be considered within several limitations. The study was conducted in a limited geographic area and may not reflect the experiences of Aboriginal women elsewhere as there is considerable variation between Aboriginal communities. However, some of the issues identified relating to the social context may be similar in other rural Indigenous communities in Australia. We focused on female smoking and perspectives, with the majority of participants being poorly educated and unemployed. Capturing the voices of this marginalised group of women is valuable. However, it is likely that more educated, employed women may have different experiences[50] and it is important that experiences of these women and of men are explored in future studies. Additionally, we were unable to recruit many non-smokers and their story is clearly important for future research. A further limitation of the present study design is that smoking initiation may have taken place a long time in the past, given that the age of our interviewees ranged from 17 to 41 years. Contemporary influences and experiences of Aboriginal girls may differ and further research to extend our insights would be valuable.

## Conclusions

Through listening to the stories of Aboriginal women and their health care providers, findings from this study provide insights into social and cultural influences concerning smoking initiation and maintenance by Aboriginal girls and women. The findings support the importance of addressing contextual factors in any strategies aimed at preventing smoking initiation or supporting cessation among Aboriginal women. Recognising the perceived benefits of smoking and finding alternative ways for young women to feel included and positive about a meaningful future are essential. This will include supporting them through school transitions and improving educational and employment opportunities. Community involvement is necessary to shift norms and expectations regarding smoking among Aboriginal people, with recognition that cigarette smoking is not part of traditional Aboriginal culture. Movement forward on this health issue will require acknowledgment of the importance of Aboriginal identity and culture as a source of strength and empowerment, while recognising the role of history, dispossession and persistent marginalization in perpetuating social and financial disadvantage and contributing to the high prevalence smoking.

## Declaration of competing interests

The authors declare that they have no competing interests.

## Authors' contributions

MP designed the study and coordinated all phases, conducted the focus groups (jointly), oversaw data management, undertook the data analysis and interpretation, and drafted the paper. JG assisted with design, data collection, interpretation of the data and editing the paper. RSF contributed to study design, data interpretation and editing the paper. All authors read and approved the final manuscript.

## Pre-publication history

The pre-publication history for this paper can be accessed here:

http://www.biomedcentral.com/1472-6874/11/55/prepub
